# The dengue virus NS1 protein; new roles in pathogenesis due to similarities with and affinity for the high-density lipoprotein (HDL)?

**DOI:** 10.1371/journal.ppat.1011587

**Published:** 2023-08-24

**Authors:** Ana C. Alcalá, Juan E. Ludert

**Affiliations:** 1 MU Center for Influenza and Emerging Infectious Diseases, University of Missouri, Columbia, MO, United States of America; 2 Department of Molecular Microbiology and Immunology, School of Medicine, University of Missouri, Columbia, MO, United States of America; 3 Bond Life Science Center, University of Missouri, Columbia, MO, United States of America; 4 Department of Infectomics and Molecular Pathogenesis, Center for Research and Advanced Studies (CINVESTAV), Mexico City, Mexico; Mount Sinai School of Medicine, UNITED STATES

## 1. Introduction

Dengue, the most important mosquito-borne viral disease transmitted to humans, is caused by any of the 4 known serotypes of the dengue virus (DENV). Dengue is a systemic disease, and clinical manifestations can range from a self-limited mild fever to a life-threatening disease. Patients with dengue fever may suffer nausea, vomiting, rash, aches, and pains, while severe dengue is characterized by plasma leakage, hemorrhages, respiratory distress, and organ failure. To date, no antivirals are available, and the use of the currently licensed vaccines is limited.

The DENV nonstructural protein 1 (NS1) is a glycoprotein (46 to 55 kDa), found as a dimer inside and on the outer phase of the cell plasma membrane [1]. NS1 is also secreted as a hexamer; yet, recent evidence obtained with recombinant NS1 (rNS1) expressed in human embryonic kidney cells indicated that in addition to hexamers, tetramers may also be secreted [2]. In the patient’s sera, NS1 peaks between 3 and 5 days after fever onset and can reach concentrations up to 50 μg/ml, which makes NS1 a useful diagnosis marker [3]. Soluble NS1 has been related to dengue pathogenesis by several different mechanisms, including complement fixation, direct disruption of tight junctions, and endothelial glycocalyx in cultured cells and activation of innate immunity via TRL4 pathways [4–6]. These NS1 properties suggest a direct role for NS1 in the coagulation disorders, endothelial plasma leakage, and the cytokine storm observed in severe dengue patients. Moreover, preexposure of vertebrate and mosquito cells to NS1 facilitates DENV replication [7–9], presumably due to the capacity of internalized NS1 to modulate cell innate immunity pathways [10]. Indeed, high levels of circulating NS1 have been associated with disease severity [11].

## 2. Dengue virus NS1 dimer is lipophilic, and the hexamer is a bona fide lipoprotein

**Fig 1:**
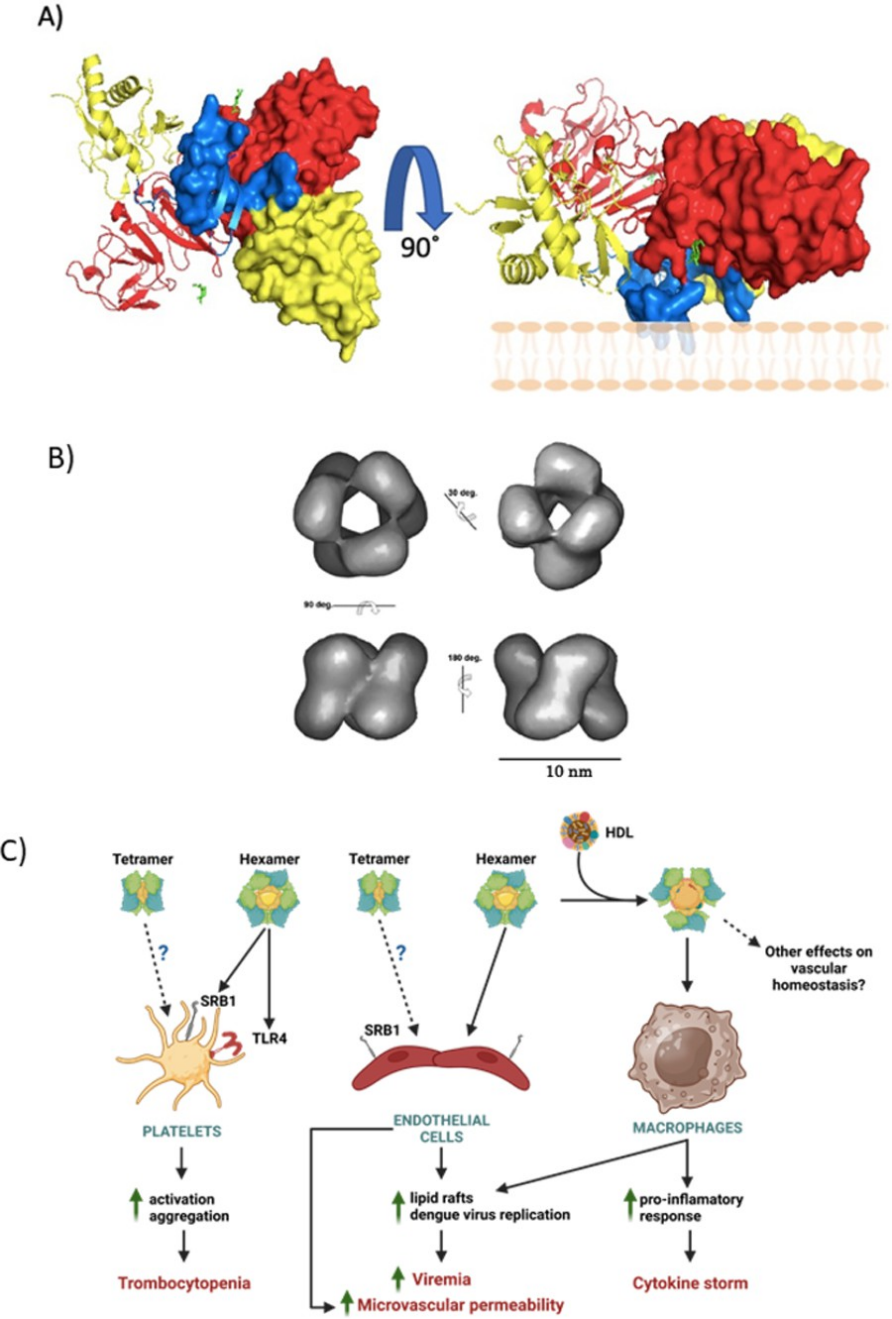
Structure of NS1 and potential novel mechanisms of pathogenesis. (**A**) NS1 dimer showing the β-roll (blue), the β-ladder (red), and wing (yellow) domains. Sugars are shown in green. One monomer is shown ribbon, and the other in surface views. NS1 images were made with Pymol based on PDB ID 4O6B. (**B**) The NS1 hexamer is shaped as an open barrel with a central channel filled with lipid molecules (lipids not shown). Hydrophobic residues, forming the greasy surface of the molecule, will phase the inside of the channel. Hexamer images taken from reference [12]. Diagram of different novel mechanisms by which NS1 may contribute to dengue pathogenesis. If circulatory forms of NS1, other than the hexamers, interact with HDL, platelets or cells is currently unknown (dashed arrows), and neither is known if other functions of HDL are altered after NS1 binding. Images were made with BioRender.

The seminal work by Gutsche and colleagues [12] showed that circulating dengue NS1 is a bona fide lipoprotein. Using cryo-electron microscopy, they showed that the NS1 hexamer is shaped as an open barrel (10 nm in diameter and 9 nm in height), with 32-point symmetry and a prominent central channel that runs along the molecule [12] (Fig 1). This structure was also evident in secreted rNS1 expressed in baculoviruses [13]. The channel is filled with triglycerides, cholesterol esters, and phospholipids. Interestingly, although the total amounts estimated for each lipid type were different, and the lipid-to-protein ratio was lower for the NS1, the composition of the soluble NS1 resembles the lipid cargo of the human high-density lipoprotein (HDL). Upon synthesis, NS1 rapidly dimerizes, acquiring increased hydrophobicity and the capacity to associate with membranes [14,15]. The resolution of the 3D structure of the dengue NS1 revealed that the dimer presents hydrophobic and hydrophilic surfaces [16]. The hydrophobic region of the monomer is located towards the amino terminus and is formed by a β-roll domain, which also participates in dimerization, and a connector loop, while the rest of the protein, comprised of the wing and ladder domains, is hydrophilic (Fig 1). In vitro, dimeric NS1 has been shown to interact and disrupt liposomes [16]; in infected cells, dimeric NS1 is found associated with internal membranes [1], as part of the replication complexes, and with lipid rafts of the plasma membrane, a property that seems to be mediated by GPI tails [17,18]. The walls of the channel, approximately 20 Å in diameter, are lined by the hydrophobic face of the 3 dimers, composed of the β-roll domains and the “greasy” fingers. Of note, while the NS1 dimer is a very stable structure, there are no bonds between the dimers in the hexamer structure, which seem to be held together, at least in part, by the hydrophobic interaction of each dimer with the lipids inside the channel [2].

But the similarities between the dengue NS1 protein and the HDL lipoprotein go beyond their lipid content. Alcala and colleagues [9] found that soluble DENV NS1 protein is capable of direct binding to the main HDL receptor, the Scavenger Receptor class B, member 1 (SRB1), with average dissociation constants in the same range as HDL, as determined by plasmon resonance assays (47.02 versus 18.71 nM for NS1 and HDL, respectively). Blocking the SRB1 in human-derived liver cells with antibodies or by HDL competition results in a significant reduction in the amount of NS1 observed inside the cells [9]. NS1 internalization is necessary to observe tight junction disruptions and p38 MAPK pathway activation in cultured cells [19,20]. However, cholesterol transfer via the SRB1 seems to take place without internalization of the HDL particle [21], and how NS1 may be internalized after attachment to the SRB1 is unknown.

The DENV NS1 protein uses nonpolar interactions to bind directly with the the Apo-A1 protein moiety of HDL [8]. In their work, using murine macrophages, Coelho and colleagues [8] described that NS1 induces an increase in lipid rafts in noninfected cells and virion attachment, thus favoring virus replication, while Apo-A1 has the opposite effect [8]. The capacity of NS1 to facilitate DENV infection is “neutralized” by Apo-A1. Thus, a way an excess of circulating NS1 may favor DENV infection is by “kidnapping” serum Apo-A1 and promoting lipid rafts exposure in target cells, including macrophages, and endothelial cells [8,22]. Finally, NS1 is capable of binding and “decorating” HDL molecules [23]. Between 2 and 4 NS1 dimers were visualized by cryo-electron microscopy bound to a single HDL molecule (3 dimers in 60% of the cases). These NS1-HDL complexes were detected in the sera collected from hospitalized dengue patients. The binding of NS1 to low-density lipoprotein (LDL) was also found but to a lesser extent. Bound dimers appear to be derived from hexamers; however, it is not clear how this latter form disassembles to interact with HDL or if the binding of NS1 to HDL molecules is Apo-A1 mediated. Interestingly, NS1-bound HDL, but not free HDL, induces a proinflammatory response in cultured human primary macrophage cells. Thus, in addition to the direct activation of cytokines secretion via TRL4 activation [6], NS1 may also contribute indirectly to the cytokine storm observed in severe dengue patients by altering HDL functions. If other functions of HDL, especially those related to endothelial protection [24] are altered upon NS1 docking is unknown. So, NS1 seems to have the capacity to bind both to the lipid ligand (HDL) as well as to the receptor (SRB1), which may be a consequence of the amphipathic character of NS1. The in vitro data obtained by surface plasmon resonance using highly purified rNS1 and SRB1 indicate that NS1 can bind directly to SRB1 [9]. Yet, in vivo, indirect binding of the NS1-HDL complex to the SRB1 receptor may also occur, making it challenging to appreciate the full structural and biological characteristics of the NS1, HDL, and SRB1 triad.

It has been documented that cholesterol is required for DENV replication and that treatment of infected cells with lipid-lowering drugs, such as statins, reduced virus replication, cytopathic effects, and virus yield [25]. However, how, or how much of this cholesterol is used for the formation, function, secretion, and oligomerization of NS1 has never been evaluated and is unknown.

## 3. Is circulating dengue virus NS1 associated with the lipid alterations observed in dengue patients?

Several studies have reported the occurrence of changes in total cholesterol levels and lipoprotein concentrations in the sera of patients with dengue. A prospective study of a 1,200-participant Nicaraguan cohort [26] showed that total and LDL and HDL cholesterol levels were decreased in dengue patients compared to other febrile illnesses, and in severe cases compared to mild cases. Lipid alterations (total cholesterol, LDL, HDL, and VLDL levels) in dengue patients have also been observed in small cohort studies [27–30]. A recent meta-analysis aimed to identify serum lipid changes as biomarkers for severe dengue [31] found an inverse correlation between total cholesterol and LDL levels, but not HDL levels, with disease severity. The demand for cholesterol by infected cells may in part be responsible for those changes. But can the circulation in sera, at quite high concentrations, of a viral protein that shares characteristics with the HDL play a role in these alterations? It is difficult to answer with the current information, but possible mechanisms will include direct competition between NS1 and HDL for the SRB1 [9], which is abundantly expressed in several tissues [32], or alterations by NS1 in the signaling pathways of the different lipoproteins particle or their metabolic turnover [23].

## 4. Can serum NS1 activate platelets, and induce thrombocytopenia, by an SRB1- and HDL-related mechanism?

Thrombocytopenia is a major laboratory finding observed in dengue patients. Platelets, together with HDL, are central players in the maintenance of vascular homeostasis and protection against vascular diseases. Several mechanisms have been proposed to explain the drop in platelet count in dengue patients, including the direct effects of NS1 and cross-reactive anti-NS1 antibodies. Chao and colleagues [33] found that dengue NS1 can directly activate platelets via TRL4, which results in aggregation, increased phagocytosis by macrophages, and augmented adhesion to endothelial cells, leading to thrombocytopenia. SRB1 is expressed on the surface of platelets and is a molecular link between HDL and platelet activation [32]. Thus, could NS1, in addition to TLR4, activate platelets via the SRB1 and cause thrombocytopenia? Recent work mapped the wing domain of NS1 as the cell binding domain [34]. The exposure of platelet-rich plasma to either full-length, β-roll domain deleted, or the isolated wing or β-ladder domain NS1 proteins also resulted in platelets activation; noticeably, the wing domain showed the highest activation capacity [35]. If NS1 binds to the SRB1 via the wind domain, or if upon binding activates any transduction pathways as HDL is unknown, and formal identification of the receptor for NS1 on platelets is necessary; yet, the current evidence suggest that NS1 may activate platelets by at least 2 different pathways, TLR4 and SRB1.

## 5. Concluding remarks

The full molecular mechanisms by which NS1 contributes to the pathogenesis of dengue are far from being understood. The new findings showing NS1 similarities with the HDL in their lipid composition and receptor usage, and the capability of NS1 to bind to the HDL particles themselves or its protein moiety Apo-A1, are exciting and suggest new ways NS1 may contribute to dengue pathogenesis, including increased virus replication, cytokine storm, and thrombocytopenia (Fig 1). Besides, they may eventually help to explain the changes in lipid homeostasis observed in dengue patients. A role for HDL and the SRB1 is being elucidated during SARS-CoV-2 infections and its complex pathogenesis [36]. However, exciting as these findings may be, many questions remain open and the underlying mechanisms need to be elucidated. Upon binding to the SRB1, does NS1 activates the same pathways as HDL, if any? Is the NS1/HDL complex still capable of binding to the SRB1? Is the binding of NS1 to Apo-A1 a prerequisite for the association with HDL? What is the participation of the wing domain in all these interactions? How does the capacity of NS1 to disrupt tight junctions relate to serum NS1 [5,37], now found to circulate in complexes with HDL? The binding of NS1 alters HDL functions, are the properties of NS1 preserved after HDL binding? Indeed, lack of association between the levels of NS1 in sera and the capacity of those sera to alter the tight junctions of cells in cultures [38], or to affect vascular leak in mice [39], has been observed. Finally, will the prevention of NS1 interaction with the SRB1 or the HDL render any clinical benefits? Hopefully, this question may soon find answers translatable to the treatment of dengue, a disease for which there is no specific antiviral treatment yet.
